# CBCT Assessment of Ethmoid Roof Variations through Keros, Gera, and TMS Classifications

**DOI:** 10.1155/2022/3708851

**Published:** 2022-08-22

**Authors:** Mina Mahdian, Mitra Karbasi Kheir

**Affiliations:** ^1^Department of Prosthodontics and Digital Technology, Stony Brook University, School of Dental Medicine, Stony Brook, NY, USA; ^2^Oral and Maxillofacial Radiologist, Private Practitioner, Isfahan, Iran

## Abstract

**Background:**

This study is designed to assess the variations of the ethmoid roof through the Keros, Gera, and Thailand-Malaysia-Singapore (TMS) classifications by means of Cone-Beam Computed Tomography (CBCT).

**Materials and Methods:**

372 CBCT scans were reviewed. The depth of the olfactory fossa (OF) was defined by the height of the lateral lamella (LL). The degree of the angle formed by the LL and the continuation of the horizontal plane passing through the cribriform plate was calculated. The risk of injury to the skull base was assessed by TMS classification.

**Results:**

The distribution of Keros types 1, 2, and 3 was 20.43%, 66.26%, and 13.31%, respectively. No significant difference was seen in the Keros classification among males and females, and asymmetry in the depth of the cribriform plate was seen in 33.3% of patients. 29.57% of the cases had type I, 61.42% had type II, and 9.01% had type III of Gera classification. 33.9% of the patients had asymmetry in the Gera classification. There was not significant difference in the Gera classification among males and females. 95.43%, 4.17%, and 0.40% of patients were of types 1, 2, and 3 of TMS classification, respectively.

**Conclusion:**

The most prevalent variations of the ethmoid roof were Keros type II, Gera type II, and TMS type 1. Although the prevalence of the dangerous types (Keros type 3, Gera type III, and TMS type 3) was low, preoperative assessment is essential in reducing surgical complications. CBCT is beneficial in evaluating these variations due to its low radiation dose.

## 1. Introduction

Functional endoscopic sinus surgery (FESS) is highly performed as the current treatment for diseases of nose and paranasal sinuses (PNS). Although there is considerable reduction in the incidence of associated complications in FESS comparing with traditional approaches, it has complications such as cerebrospinal fluid leak, orbital hurt, and intracranial injury [1–3].

The olfactory fossa (OF) is a depression in the anterior cranial cavity. The cribriform plate (CP) of ethmoid forms its floor. This delicate bony plate separates the anterior cranial fossa from the nasal cavity. Lateral and medial limitations of OF are lateral lamella (LL) of cribriform plate and crista galli, respectively [4]. OF contains the olfactory bulbs and tracts. The lateral boundary of the CP of the ethmoid bone is called lamina lateralis (LL). It runs vertically and joins the fovea ethmoidalis inferomedially. The LL is the thinnest bone in the anterior skull base offering lowest resistor to perforation during surgical maneuvers. Hence, it is a high-risk site where eventually surgical complications occur [5, 6]. If one of the lateral lamellae is inferiorly positioned, reporting should be considered, as this increases the risk of intraoperative injury. Therefore, asymmetry in the OF depth or the height of LL is related with higher risk of intracranial penetration during surgeries such as FESS.

### 1.1. Keros Classification

The height of lateral lamella is assessed by the depth of the olfactory fossa (Keros classification). In 1962, Keros classified the depth of the OF into Keros type I (<3 mm), type II (4–7 mm) and type III (8–16 mm) [7]. The Keros type III is the most susceptible one, considering the major risk for iatrogenic injury of the LL of the CP [8].

### 1.2. Gera Classification

It is the degree of the angle at which the lateral lamella joints on the cribriform plate show variations among patients. Also, the sloping shape of the skull causes limitations in the Keros classification in terms of defining the risk of intracranial penetration. Therefore, the Gera classification was introduced to consider the sloping shape of ethmoid roof (ER) relative to CP by measuring the angle formed by the LL of CP and the continuation of the horizontal plane passing through CP. The risk of intracranial entry was divided into 3 classes: class I (>80 degrees, low risk), class II (45 to 80 degrees, medium risk), and class III (<45 degrees, high risk) [9, 10].

### 1.3. TMS Classification

The Thailand-Malaysia-Singapore (TMS) Classification was proposed as a new practical radiological classification that completes the Keros and Gera classifications and assesses the anatomical risk of anterior skull base injury by means of the orbital floor as a reference. The distances from orbital floor to cribriform plate (OFL-CP) and from orbital floor to ethmoid roof (OFL-ER) were classified into type 1, type 2, and type 3 [11].

Radiographic analysis using multidetector computed tomography (MDCT) has been supposed to be the gold standard in the presurgical evaluation of the PNS, which provides a knowledge of the anatomic variations of the ethmoid roof in every patient. Cone beam computed tomography (CBCT) is widely applied in dentistry and otorhinolaryngology for analysis of PNS due to its low radiation exposure and high-resolution images. Variations of ethmoid roof have been assessed using the Keros, Gera, and TMS classifications on MDCT images. However, there are a few studies in the literature that have used CBCT images to assess those anatomic variations [1–33]. This study is clinically significant as it can show the ability of CBCT devices to show the anatomic variations of the ethmoid roof in the preoperative examinations of patients, while its radiation dose is far less than that of MDCT. So, this study is designed to assess the variations of ethmoid roof through the Keros, Gera, and TMS classifications using CBCT images.

## 2. Materials and Methods

### 2.1. Sample Selection

372 CBCT scans from 900 images were, retrospectively, retrieved from database of the department of oral and maxillofacial radiology at Isfahan University of Medical Sciences School of Dentistry. The Human Research Ethics Committee of Isfahan University of Medical Sciences reviewed and authorized the protocol of the study (Ethical Approval Code: 293070), which was performed under the Declaration of Helsinki and approved guidelines.

CBCT scans were obtained using Sirona Orthophos, GALILEOS version 1.7, (Sirona, Germany) with a flat panel detector. The selected scan parameters were 85 kVp and 10–42 mA, according to the size of patients. The exposure time was 14 seconds, the effective exposure time was 2–6 seconds, and the voxel size was 0.3 *∗* 0.3 *∗* 0.3 mm.

For choosing proper images, each of the 900 CBCT scans were opened on a computer viewer (E2042C, Korea) through Galileos software, and the panorama view was observed. Then, all of the coronal sections in the PNS area were scrolled, and the inclusion and exclusion criteria were checked in panorama and coronal sections of each CBCT scan.

The inclusion criteria consisted of scans of patients with completely developed PNS, acquired for any reason (i.e., implant treatment planning, impacted teeth, orthodontic treatment planning, etc.) showing the crista galli of the ethmoidal bone and nasal fossa, with no gender consideration.

Patients with a history of paranasal sinus surgery, maxillofacial trauma, and pathological processes in the PNS were excluded from the study. Also, low-quality images, images containing only lower jaw, and images containing artifacts, which made visualization of anatomical structures difficult, were excluded from the evaluation. Finally, based on the above criteria, 372 CBCT scans were selected for the study.

### 2.2. Image Analysis

All of the 372 CBCT scans were evaluated by one oral and maxillofacial radiologist with experience in CBCT imaging twice, with a month interval on an LG LED computer viewer (E2042C, Korea) by means of Galileos software.

The following anatomical landmarks were identified on coronal sections and used for measurements:

1. Cribriform plate (CP)
2. Lateral lamella of cribriform plate (LL)
3. Medial ethmoidal roof point (ER), (medial end of ethmoid roof articulating with LL)
4. Orbital floor (OFL)
5. Olfactory fossa (OF).

### 2.3. Keros Classification

**Figure 1:**
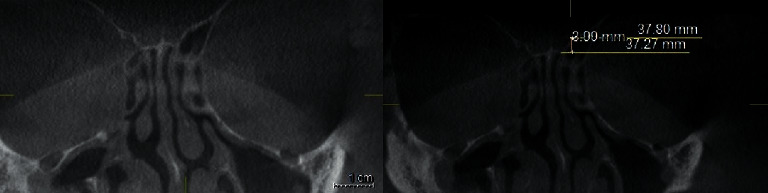
Coronal plan of CBCT demonstrating type 1 Keros classification on the left side.

**Figure 2:**
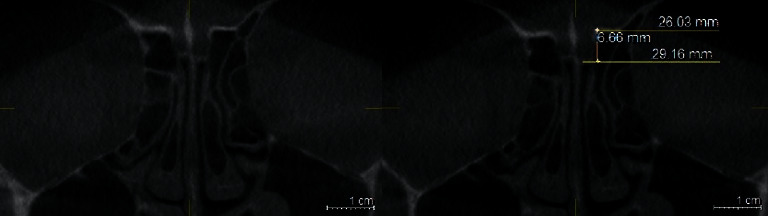
Coronal plan of CBCT demonstrating type 2 Keros classification on the left side.

**Figure 3:**
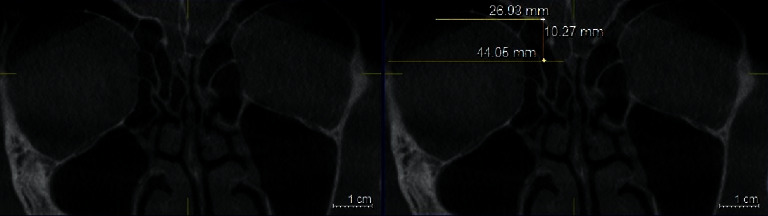
Coronal plan of CBCT demonstrating type 3 Keros classification on the right side.

The depth of the OF was defined by the height of the LL on the coronal sections and at the level of infraorbital foramina. The linear measurements (in millimeters) were done using the Galileos software's ruler tool for both lateral lamellae of the CP (right and left sides). Horizontal lines were drawn along the CP and at the ER. The vertical interval between these two horizontal lines was considered as the height of LL (Figures 1–3).

OF depth was classified into three categories: [7].

- Type 1: has a depth of 1–3.9 mm (low risk)
- Type 2: has a depth of 4–7.9 mm (medium risk)
- Type 3: has a depth of 8–16 mm (high risk).

The type of OF depth was determined for each side of a patient and the difference between the right and left side, considered as asymmetric LL.

### 2.4. Gera Classification

**Figure 4:**
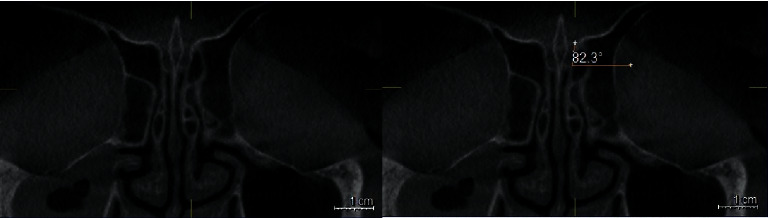
Coronal plan of CBCT demonstrating type I Gera classification on the left side.

**Figure 5:**
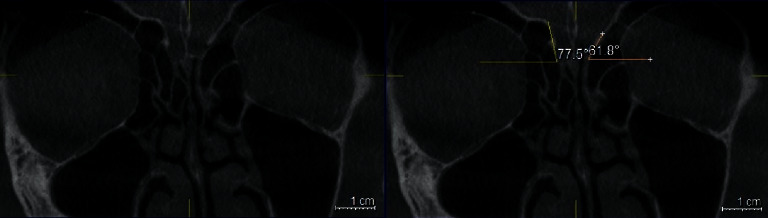
Coronal plan of CBCT demonstrating type II Gera classification on both sides.

**Figure 6:**
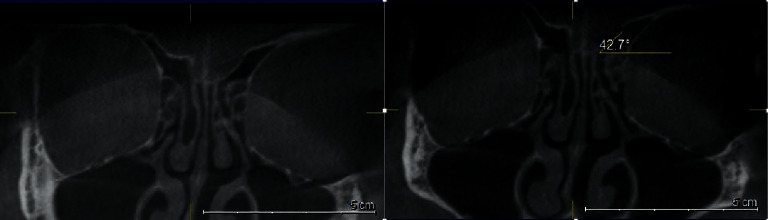
Coronal plan of CBCT demonstrating type III Gera classification on the left side.

The degree of the angle formed by the LL and the continuation of the horizontal plane passing through the CP was calculated using the Galileos software's measure-angle tool for each side. According to Gera system, the angle is classified depending on its degree and on the hypothetical risk of iatrogenic injuries (Figures 4–6) [9].

- Class I (>80 degrees, low risk)
- Class II (45 to 80 degrees, medium risk)
- Class III (<45 degrees, high risk).

The difference between the right and left sides was considered as asymmetry in the position of LL.

### 2.5. TMS Classification

**Figure 7:**
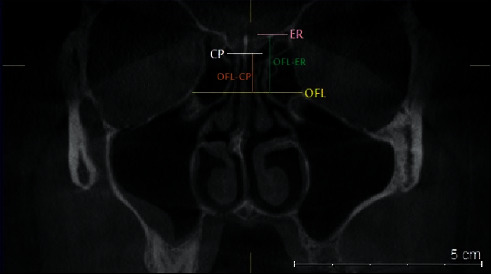
Coronal plan of CBCT demonstrating landmarks of TMS classification: orbital floor (OFL): yellow line, cribriform plate (CP): white line, ethmoid roof (ER): pink line, OFL-CP: orange line, OFL-ER: green line.

In each side, the orbital floor was defined at the point where the medial wall of the maxillary sinus roof was observed at its maximum height. The distances from OFL to CP (OFL-CP) and from OFL to ER (OFL-ER) were used in each side using the Galileos software's ruler tool. Horizontal lines were drawn along the CP and at the OFL and ER. The vertical interval between OFL and CP horizontal lines was considered as the OFL-CP distance, and the vertical distance between OFL and ER horizontal lines was considered as the OFL-ER distance (Figure 7).

**Figure 8:**
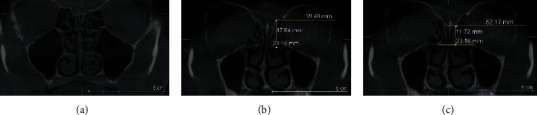
(a) Coronal plan of CBCT demonstrating type 1 TMS classification on the left side. (b) OFL-ER measurement and (c) OFL-CP measurement coronal plan of CBCT.

**Figure 9:**
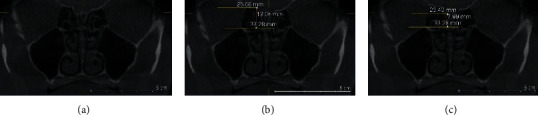
(a) Coronal plan of CBCT demonstrating type 2 TMS classification on the right side. (b) OFL-ER measurement and (c) OFL-CP measurement coronal plan of CBCT.

**Figure 10:**
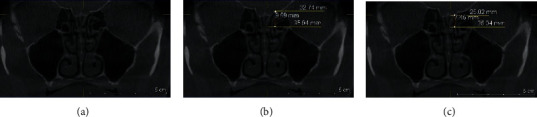
(a) Coronal CBCT demonstrating type 3 TMS classification on the left side. (b) OFL-ER measurement and (c) OFL-CP measurement coronal plan of CBCT.

The risk of skull base injury was classified into three groups as follows (Figures 8–10) [11]:

- Type 1 (low risk) is when both OFL-CP and OFL-ER are 10 mm and above, or more than twice the depth of thru-cutting forceps when ESS is supposed to be safe.
- Type 2 (moderate risk) is when either OFL-CP or OFL-ER is less than 10 mm, or less than twice the depth of thru-cutting forceps when ESS should go forward cautiously.
- Type 3 (high risk) is when both OFL-CP and OFL-ER are less than 10 mm or less than the depth of thru-cutting forceps when ESS should proceed very cautiously.

### 2.6. Statistical Analysis

Descriptive parametric data were offered as percentage. The Kappa coefficient was calculated for the agreement between the data obtained from the first and second observations in each classification. Wilcoxon signed ranks test was applied to assess the difference in scores of each classification on the right and left side. The *U* Mann-Whitney test was used to assess the difference of classification scores in gender. The correlations between TMS, Keros, and Gera classifications were estimated using Pearson's correlation coefficient (*r*). The statistical significance was set to *P* < 0.05.

## 3. Results

### 3.1. Demographic Data

CBCT scans of 372 subjects were contained in the study. Among 372 subjects, 195 were males (52.4%) and 177 were females (47.6%). The youngest subject was 14 years, and the eldest was of 96 years of age, with a mean age of 41 and a mode age of 19.

### 3.2. Keros Classification

The Kappa coefficient for the agreement between the first and second observations of Keros classification was 0.978 (*P*-value = 0.00) in the left side and 0.984 (*P*-value = 0.00) in the right side. Due to the high agreement between the first and second observations, the data of the first observation was used in statistical analyzes.

Table 2 shows the distribution of keros scores according to the side.

Table 1 presents the distribution of Keros scores according to their sides and sex. The *U* Mann-Whitney test showed no significant statistical difference in the distribution of the Keros scores among male and female.

The Kappa coefficient for the agreement of Keros scores between the right and left side was 0.336 (*P*-value = 0.00).  Table 3 shows that 66.7% of images had similar Keros scores, and 33.3% of the images had different Keros scores in the right and left sides. The Wilcoxon signed ranks test showed a significant difference between the Keros scores of the right and left side (*P*-value = 0.03).

### 3.3. Gera Classification

The Kappa coefficient for the agreement between the first and second observations of Gera classification was 0.945 (*P*-value = 0.00) in the left side and 0.926 (*P*-value = 0.00) in the right side. Due to the high agreement between the first and second observations, the data of the first observation was used in statistical analyses.

Table 2 shows the distribution of Gera scores according to the side.

Table 1 presents the distribution of Gera scores according to their sides and sex. The *U* Mann-Whitney test showed no significant statistical difference in the distribution of the Gera scores among male and female.

The Kappa coefficient for the agreement of Gera scores between the right and left side was 0.361 (*P*-value = 0.00).  Table 4 shows that 66.1% of images had similar Gera scores, and 33.9% of the images had different Gera scores in the right and left sides. The Wilcoxon signed ranks test showed a significant difference between the Gera scores of the right and left sides (*P*-value = 0.011).

### 3.4. TMS Classification

The Kappa coefficient for the agreement between the first and second observations of TMS classification was 0.967 (*P*-value = 0.00) in the left side and 0.973 (*P*-value = 0.00) in the right side. Due to the high agreement between the first and second observations, the data of the first observation was used in statistical analyzes.

Table 2 shows the distribution of TMS scores according to the side.

Table 1 presents the distribution of TMS scores according to their sides and sex. The *U* Mann-Whitney test showed a significant statistical difference between gender and TMS scores of the left side (*P*-value = 0.041).

The Kappa coefficient for the agreement of TMS scores between the right and left side was 0.571 (*P*-value = 0.00).  Table 5 shows that 96.2% of images had similar TMS scores and 3.8% of the images had different TMS scores in the right and left sides. The Wilcoxon signed ranks test showed no significant difference between the TMS scores of the right and left sides (*P*-value = 0.467).

### 3.5. Comparison of Skull Base Classifications

The correlation between Gera, TMS, and Keros classifications was assessed (Table 6). Table 6 showed a weak negative correlation between Gera and Keros classifications in the right and left sides (*P*-value = 0.00) and a positive correlation between the right and left sides in each classification (*P*-value = 0.00). There was no significant correlation between age and Gera, Keros, and TMS classifications (Table 6).

## 4. Discussion

FESS is a common method for the management of PNS disease. LL, which is the lateral boundary of the CP of the ethmoid bone, is considered a high risk site where most surgical complications occur. It may be prominent and protrude into the anterior ethmoid sinus. The LL may be harmed during operations, specifically when it protrudes into the sinus cavity. LL is susceptible to surgical trauma when the disease within the frontal recess along the superolateral wall of the middle turbinate is removed, particularly when using biting forceps. Thus, a great attention to the LL of the CP plate is required during endoscopic ethmoid and frontal sinus surgeries to prevent potential complications [5, 6].

### 4.1. Keros Classification

Traditionally, Keros classification has been used to categorize OF depth of the CP as an index of risk for skull base entry during FESS. The depth of the OF was determined by the height of the LL [7].

In this study, the distribution of Keros types 1, 2, and 3 was 20.43%, 66.26%, and 13.31%, respectively.  Table 7 summarized the incidence of Keros types in different populations and showed that most studies were done using MDCT, and only two studies have been performed using CBCT so far.

About the distribution of Keros types, type 2 was the most frequently viewed in this survey, which was similar to the majority of previous reports [1, 3, 4, 7, 8, 10, 11, 13, 14, 16–19, 22–24, 26–28, 30–32]. The most similar one to this study is the costa et al.'s study, which was done on Brazilian population using CBCT. They have reported a frequency of 65.52 for Keros type 2 [13].

This study showed that Keros type 3 had the lowest distribution among the Iranian population, and the distribution of Keros type 1 was more than Keros type 3, while, in the articles of costa et al. and Guldner et al. using CBCT, with different sample size, the distribution of Keros type 3 was more than type 1, which might be due to the ethnical differences. Keros type 3 patients have been reported to be more susceptible to iatrogenic skull-base injuries [13, 18, 29].

This study showed no significant difference in the Keros classification among males and females, which is in accordance with some studies [9, 13, 14, 24]. However, the distribution of Keros type between two genders is reported to be different in some other studies [1, 3, 13].

Asymmetry in the depth of the cribriform plate was seen in 33.3% of patients of the present study, which was different with 26% of Abdullah et al.' study, 75% of Babu et al.'s study, 94.8% of Adeel et al.'s study, 11.7% Nair's study, 12.3% Gera et al.'s study, and 14.6% Ali et al.'s study [1, 3, 9, 13, 25, 30]. No significant difference is reported in the distribution of Keros type between the left and right sides in some studies [12, 13, 24]. Considering the presence of asymmetry in many patients, preoperative assessments are required through MDCT or CBCT to avoid the risk of intracranial penetration during endoscopic sinus operation.  Table 7 summarized the incidence of Keros types in different populations.

### 4.2. Gera Classification

Gera et al. introduced another classification due to the limitations of Keros classification in 2018. According to this classification, a more pronounced slop of the anterior skull base on the coronal plane or the Gera type III may predispose to iatrogenic skull base injuries [9]. By using Gera classification in this study, 29.57% of our CBCT cases had type I (low risk), 61.42% had type II (moderate risk), and 9.01% had type III (high risk). The distribution of Gera classification of this study was similar to the previous reports [9, 11].

Table 8 summarized the incidence of Gera types in different populations and showed that all of the studies were done using CT, and there was no study using CBCT so far.

Preti et al. compared Keros classification with Gera classification on CT scans of 124 patients. They concluded that Gera classification showed higher sensitivity, specificity values on preoperative CT scan risk evaluation and therefore suggested to use it in combination with keros classification to prevent injuries during FESS [10].

Asymmetry in the Gera classification was found in 33.9% of the patients of this study. None of previous studies have reported asymmetry between the two sides in Gera classification [9, 11].

This study showed no significant difference in the Gera classification between two genders, which is in accordance with other studies [9, 11].

The present study found a weak negative correlation between Gera and Keros classifications. Similarly, Gera et al. reported a negative correlation between them, while Abdullah et al. presented a weak positive correlation between them. Anyway, both studies have suggested to use both classifications for the anterior skull base injuries assessments [11].

### 4.3. TMS Classification

Abdullah et al. introduced the TMS classification using CT scans in 2020 to determine the risk of skull base damage preoperatively and intraoperatively. They concluded that the combination of TMS classification with Keros and Gera classifications provided a superior intracranial injury risk assessment before and during FESS [11].

In the present study, using CBCT, 95.43% had type 1 (low risk), 4.17% had type 2 (moderate risk), and 0.40% had type 3 (high risk) according to TMS classification. There was a significant difference in the distribution of the TMS scores between two genders on the left side (*P*-value = 0.041), and the frequency of each TMS scores was higher in females. The right and left side asymmetry in the TMS scores was not significant.

TMS type 1 was the most current viewed in this study, which was close to Abdullah et al.'s findings. However, the distribution of TMS types 2 and 3 in the present study differed in that TMS type 3 was reported to be more frequent than type 2 in their study, which might be due to our greater sample size or ethnic differences. Moreover, the present study showed a significant difference in the distribution of the TMS scores between two genders on the left side, while such difference was not reported in Abdullah et al.'s study [11].

No significant correlation was observed between Gera and TMS classifications in this study, which was in accordance with Abdullah et al.'s findings. However, Abdullah et al.'s study reported a significant correlation between TMS and Keros classifications, which was not observed in the present study [11].

## 5. Conclusion

The most common type of OF in the study population was Keros type II. The most prevalent degree of the angle formed by the LL and the continuation of the horizontal plane passing through the CP was 45 to 80 degree (type II) according to Gera classification. The majority of our cases showed low risk for skull base injury (TMS type 1). Even though the prevalence of the high-risk types (Keros type 3, Gera type III, and TMS type 3) was low, preoperative assessment is essential to reduce surgical complications. Due to the low dose of radiation in CBCT, it is beneficial to evaluate the anterior skull base variations.

## Figures and Tables

**Table 1:** The distribution of Keros, Gera, and TMS scores according to the sides and gender.

Variables			Gender	
			Male	Female
Keros scores/right side	1	Count	49	35
		% within gender	25.1%	19.8%
	2	Count	122	121
		% within gender	62.6%	68.4
	3	Count	24	21
		% within gender	12.3%	11.9%

Keros scores/left side	1	Count	38	30
		% within gender	19.5%	16.9%
	2	Count	129	121
		% within gender	66.2%	68.4%
	3	Count	28	26
		% within gender	14.4%	14.7%

Gera scores/right side	I	Count	49	47
		% within gender	25.1%	26.6%
	II	Count	126	115
		% within gender	64.6%	65%
	III	Count	20	15
		% within gender	10.3%	8.5%

Gera scores/left side	I	Count	63	61
		% within gender	32.3%	34.5%
	II	Count	114	102
		% within gender	58.5%	57.6%
	III	Count	18	14
		% within gender	9.2%	7.9%

TMS scores/right side	1	Count	189	164
		% within gender	96.9%	92.7%
	2	Count	5	13
		% within gender	2.6%	7.3%
	3	Count	1	0
		% within gender	0.5%	0%

TMS scores/left side	1	Count	191	166
		% within gender	97.9%	93.8%
	2	Count	4	9
		% within gender	2.1%	5.1%
	3	Count	0	2
		% within gender	0%	1.1%

**Table 2:** The distributions of Keros, Gera, and TMS classification scores according to the side.

Variables	Right side		Left side		Total	
	*N*	Percentage	*N*	Percentage	*N*	Percentage
Keros score 1	84	22.6	68	18.3	152	20.43
Keros score 2	243	65.3	250	67.2	493	66.26
Keros score 3	45	12.1	54	14.5	99	13.31
Total	372	100	372	100	744	100

Gera score I	96	25.8	124	33.3	220	29.57
Gera score II	241	64.8	216	58.1	457	61.42
Gera score III	35	9.4	32	8.6	67	9.01
Total	372	100	372	100	744	100

TMS score 1	353	94.9	357	96	710	95.43
TMS score 2	18	4.8	13	3.5	31	4.17
TMS score 3	1	0.3	2	0.5	3	0.40
Total	372	100	372	100	744	100

**Table 3:** Keros scores in the right and left sides.

Variables			Keros scores right side		
			1.00	2.00	3.00
Keros scores left sides	1.00	Count	40	28	0
		% of total	10.8%	7.5%	0.0%
	2.00	Count	41	186	23
		% of total	11.0%	50.0%	6.2%
	3.00	Count	3	29	22
		% of total	0.8%	7.8%	5.9%

**Table 4:** Gera scores in the right and left sides.

Variables			Gera scores right side		
			I	II	III
Gera scores left sides	I	Count	64	55	5
		% of total	17.2%	14.8%	1.3%
	II	Count	30	169	17
		% of total	8.1%	45.4%	4.6%
	III	Count	2	17	13
		% of total	0.5%	4.6%	3.5%

**Table 5:** TMS scores in the right and left sides.

Variables			TMS scores right side		
			1.00	2.00	3.00
TMS scores left sides	1.00	Count	349	7	1
		% of total	93.8%	1.9%	0.3%
	2.00	Count	4	9	0
		% of total	1.1%	2.4%	0.0%
	3.00	Count	0	2	0
		% of total	0.0%	0.5%	0.0%

**Table 6:** Pearson's correlation coefficient between variations.

Classification scores	Pearson's correlation coefficient
Keros left with keros right	0.467 (*P*-value = 0.00)
Keros left with Gera left	−0.327 (*P*-value = 0.00)
Keros left with Gera right	−0.293 (*P*-value = 0.00)
Keros left with TMS left	0.012 (*P*-value = 0.819)
Keros left with TMS right	−0.027 (*P*-value = 0.599)
Keros left with age	0.028 (*P*-value = 0.592)
Keros right with Gera left	−0.241 (*P*-value = 0.00)
Keros right with Gera right	−0.301 (*P*-value = 0.00)
Keros right with TMS left	0.082 (*P*-value = 0.116)
Keros right with TMS right	−0.022 (*P*-value = 0.666)
Keros right with age	−0.033 (*P*-value = 0.528)
Gera left with Gera right	0.434 (*P*-value = 0.00)
Gera left with TMS left	0.039 (*P*-value = 0.458)
Gera left with TMS right	−0.010 (*P*-value = 0.853)
Gera left with age	−0.048 (*P*-value = 0.355)
Gera right with TMS left	−0.012 (*P*-value = 0.811)
Gera right with TMS right	−0.039 (*P*-value = 0.450)
Gera right with age	−0.038 (*P*-value = 0.463)
TMS left with TMS right	0.635 (*P*-value = 0.00)
TMS left with age	−0.077 (*P*-value = 0.140)
TMS right with age	−0.069 (*P*-value = 0.183)

**Table 7:** The incidence of Keros types in different populations.

Study	Country	Sample number	Modality	Keros type 1 (%)	Keros type 2 (%)	Keros type 3 (%)
Keros [7]	Germany	450	Cadaver	12	70	18
Babu et al. [1]	India	1200	CT	17.5	74.6	7.9
Solares et al. [15]	USA	50	CT	83	15	2
Gauba et al. [21]	UK	32	CT	34.4	28.1	37.5
Floreani et al. [22]	Australia	22	CT	23	50	27
Souza et al. [8]	Brazil	200	CT	26.3	73.3	0.5
Paber et al. [12]	Philippines	218	CT	81.6	17.9	0.5
Nitinavakarn et al. [23]	Thailand	176	CT	11.9	68.8	19.3
Salroo et al. [24]	India	100	CT	29	61	10
Pawar et al. [27]	India	200	CT	18.5	74.5	7
Satish Nair [30]	India	180	CT	17.2	77.2	5.6
Ali et al. [25]	India	75	CT	20	78.7	1.3
Gupta et al. [26]	India	100	CT	39	59	2
Jacob et al. [4]	India	32	Dry skull	23.44	70.8	5.73
Preti et al. [10]	Italy	124	CT	29.03	59.68	11.29
Bista et al. [20]	Nepal	50	CT	86	12	2
Adeel et al. [3]	Pakistan	77	CT	29.9	49.4	20.8
Anderhuber et al. [16]	Germany	272	CT	14.2	70.6	15.2
Basak et al. [17]	Turkey	64	CT	9	53	38
Jang et al. [28]	South Korea	205	CT	30.5	69.5	Nil
Abdullah et al. [11]	Malaysia	150	CT	24.3	75.7	Nil
Goytia et al. [31]	México	100	CT	17	63	20
Leunig et al. [19]	Germany	641	CT	8	80	12
Elwany et al. [32]	Egypt	300	CT	42.5	56.8	1.4
Alazzawi et al. [33]	Malaysia	150	CT	80	20	Nil
Kaplanoglu et al. [14]	Turkey	500	CT	13.4	76.1	10.5
Costa et al. [13]	Brazil	174	CBCT	13.79	65.52	20.69
Guldner et al. [18]	Germany	865	CBCT	17.3	58.6	24.1

**Table 8:** The incidence of gera types in different populations.

Study	Country	Sample number	Modality	Gera type I (%)	Gera type II (%)	Gera type III (%)
Gera et al. [9]	Italy	190	CT	32.6	62.7	4.7
Abdullah et al. [11]	Malaysia	150	CT	23.7	72.3	4

## Data Availability

The data used to support the findings of this study are available from the corresponding author upon request.

## Abbreviations

TMS: Thailand-Malaysia-Singapore
CBCT: Cone-beam computed tomography
OF: Olfactory fossa
LL: Lateral lamella
FESS: Functional endoscopic sinus surgery
PNS: Paranasal sinuses
CP: Cribriform plate
ER: Ethmoid roof
OFL: Orbital floor
MDCT: Multidetector computed tomography.
